# Stratified meta-analysis by ethnicity revealed that *ADRB3* Trp64Arg polymorphism was associated with coronary artery disease in Asians, but not in Caucasians

**DOI:** 10.1097/MD.0000000000018914

**Published:** 2020-01-24

**Authors:** Yingjian Chen, Yuanjun Liao, Shengnan Sun, Fan Lin, Rang Li, Shujin Lan, Xiaolei Zhao, Jiheng Qin, Shaoqi Rao

**Affiliations:** a School of Public Health; b Institute of Medical Systems Biology, Guangdong Medical University, Dongguan, China.

**Keywords:** β_3_-adrenergic receptor, association, coronary artery disease, polymorphism, stratified meta-analysis

## Abstract

**Background:**

Previous studies demonstrated that *ADRB3*, beta-3 adrenergic receptor, participated in lipolysis and thermogenesis in adipose tissue. Consequently, this gene has attracted an increasing number of genetic studies examining its association with coronary artery disease (CAD) in different ethnicities in recent years, but no conclusion has been reached so far. The aim of this study was to explore whether the well-studied locus *ADRB3* Trp64Arg in this gene confers a race-specific effect to CAD by conducting a stratified meta-analysis involving 15 independent studies and 11,802 subjects.

**Methods:**

Odds ratios (ORs) and 95% confidence intervals (CIs) were calculated to assess the strength of association. Publication bias was quantified and examined with Begg's funnel plot test and Egger's linear regression method. The overall meta-analysis or stratified meta-analysis by ethnicity was performed by using STATA 12.0 software.

**Results:**

A total of 15 eligible studies involving 5779 CAD cases and 6023 health controls were included in this meta-analysis. The pooled results indicated that *ADRB3* Trp64Arg polymorphism was significantly associated with an increased risk of CAD. Further stratified analysis by ethnicity revealed that *ADRB3* Trp64Arg polymorphism was significantly associated with CAD in Asians (allelic: OR = 1.48, 95%CI 1.13–1.94, *P* = .005; homozygous: OR = 2.66, 95%CI 1.87–3.77, *P* < .001; recessive: OR = 2.46, 95%CI 1.74–3.47, *P* < .001), but not in Caucasians (allelic: OR = 1.09, 95%CI 0.93–1.27, *P* = .290; homozygous: OR = 1.31, 95%CI 0.61–2.86, *P* = .490; recessive: OR = 1.31, 95%CI 0.60–2.84, *P* = 2.494).

**Conclusions:**

This meta-analysis suggests that *ADRB3* Trp64Arg polymorphism confers a race-specific effect to CAD.

## 1 Introduction

Coronary artery disease (CAD) has become the most prevalent cardiovascular disease, which is one of the diseases with high mortality and has been recognized as the first killer of human health.^[1]^ The etiology of CAD is multifactorial, involving a large number of genes and environmental factors. It has been reported that its disease susceptibility involves a list of genetic polymorphisms.^[2,3]^ In the past decades, the polymorphisms of several susceptibility genes for CAD (e.g., *ApoE*, *ApoB*, *NOS3*, *ACE*) have been well examined by independent studies or a pooled analysis. In this study, we aimed to systematically assess its properties of the association of the β_3_-adrenergic receptor (*ADRB3*) gene polymorphism Trp64Arg with the risk of CAD in different ethnic backgrounds.

*ADRB3* is located mainly in adipose tissue and is involved in the regulation of lipolysis and thermogenesis. A previous human study found that it is closely related to obesity,^[4]^ which in turn can lead to insulin resistance and the end point events of insulin resistance syndrome including stenocardia, myocardial infarction, hypertension and/or other complications.^[5]^*ADRB3* Trp64Arg (rs4994) is a single nucleotide polymorphism (SNP) locus^[6]^ and its missense variation results in a change of amino acids (from tryptophan to arginine), thus altering the conformation of the first intracellular loop and possibly the movement of the receptor to the cell surface and G-protein binding.^[7,8]^ Several studies^[7,9]^ revealed that disruption in the signal transduction pathway may result in reduced *ADRB3* activity, rendering that *ADRB3* may play a significant role in controlling energy expenditure through the regulation of lipolysis and thermogenesis. Consequently, *ADRB3* has been deemed to a hot candidate for genetic association with CAD.

Up to date, more than 10 studies have been performed to examine the relationship between *ADRB3* Trp64Arg polymorphisms and CAD in different racial populations, but no consensus regarding this locus has been reached. There are several reasons that may explain these inconsistent results, for examples, genetic heterogeneities across different ethnicities and limited sample sizes that can be provided by individual studies. Therefore, further large-scale stratified meta-analysis to explore the properties of the association of the *ADRB3* Trp64Arg locus with the risk of CAD in different ethnic backgrounds is highly demanding.

## 2 Materials and methods

### 2.1 Literature search

Major electronic literature databases were systematically searched, which included PubMed, EMBASE, Wanfang, China National Knowledge Infrastructure (CNKI) database, and SinoMed database, up to September 2018, for all publications about the association of *ADRB3* Trp64Arg polymorphism with CAD. With the purpose of getting as many potentially relevant publications, the following keywords were used in the search: “β_3_-AR or *ADRB3* or rs4994 or C190T or Trp64Arg or β_3_-adrenergic receptor” and “polymorphism or genetics or mutation or variation or variant” and “CAD or CHD or coronary heart disease or coronary artery disease or ischemic heart disease or myocardial infarction or MI.” The references of previous meta-analyses and reviews were also manually searched to identify other studies.

### 2.2 Inclusion and exclusion criteria

To select eligible studies in this meta-analysis, the following criteria for inclusion were defined:

(i) studies aiming to assess the associations between *ADRB3* Trp64Arg polymorphism and the CAD or MI;
(ii) complete genotype data were available;
(iii) case–control or cohort studies.

If there were multiple publications from the same study group, the most recent study was included in this meta-analysis. However, if the study was a review, lecture, editorial, correspondence letter, or without full text (e.g., abstracts, meeting reports), it was excluded. Also, Hardy–Weinberg equilibrium (HWE) was checked for each study and the studies whose healthy control groups failed HWE (*P* < .01) were also excluded.^[10]^

### 2.3 Data extraction

Data of eligible studies were collected by 2 reviewers independently with a standard data collection form, which included following information:

(i) the first author's name;
(ii) the year of publication;
(iii) the country and ethnicity of the participants;
(iv) genotyping method;
(v) the frequency of genotypes.

Then, a group discussion was conducted to remedy any discrepancies in data collected by different reviewers.

### 2.4 Quality assessment

The quality of the eligible studies included in the meta-analysis was assessed by using Newcastle-Ottawa Scale (NOS) criteria,^[11]^ which is a star rating system. The NOS contains 8 items, categorized into 3 dimensions including selection, comparability and exposure. The NOS ranges between 0 and 9 stars. A full score is 9 stars and a score range of 5 to 9 stars is considered to be high quality while a score range 0 to 4 is considered to be a poor quality.^[12]^ Conflicting evaluations on the NOS score of the studies were resolved through a comprehensive reassessment by 2 reviewers.

### 2.5 Statistical analysis

This meta-analysis was performed with STATA statistical software (Version 12.0). Five genetic models were used to evaluate the association between *ADRB3* Trp64Arg polymorphism and CAD: the allelic, homozygous, heterozygous, dominant, and recessive model, respectively. In order to assess the general or race-specific effect of this polymorphism locus, both pooled and stratified meta-analysis by ethnicity (Asian, Caucasian, and African ancestry) were performed. The odds ratios (ORs) and 95% confidence intervals (CIs) were calculated to assess the strength of the association between this polymorphism locus with CAD. The significance of OR was determined with the *Z*-test, and *P* < .05 was regarded as statistically significant.^[13]^ Heterogeneity among studies was examined with *χ*^2^-based Q statistic.^[14]^ If there was statistically significant heterogeneity among studies (*P* < .05 or *I*^2^ > 50%), a random effect model (Dersimonian-Laird method) was selected to merge data. Otherwise, a fixed effect model (Mantel-Haenszel method) was employed to analyze data.^[15]^ Then, publication bias was estimated by Begg's funnel plot test and Egger's linear regression test.^[16,17]^ Finally, sensitivity analysis was performed to assess the influence of each individual study by omitting the individual study each round and calculating pooled ORs again. If the exclusion of any individual study did not alter the meta-analysis result, it indicated that the outcomes were robust.^[18]^ This study was approved by the Ethics Committee of Guangdong Medical University.

## 3 Results

### 3.1 Characteristics of eligible studies

**Figure 1:**
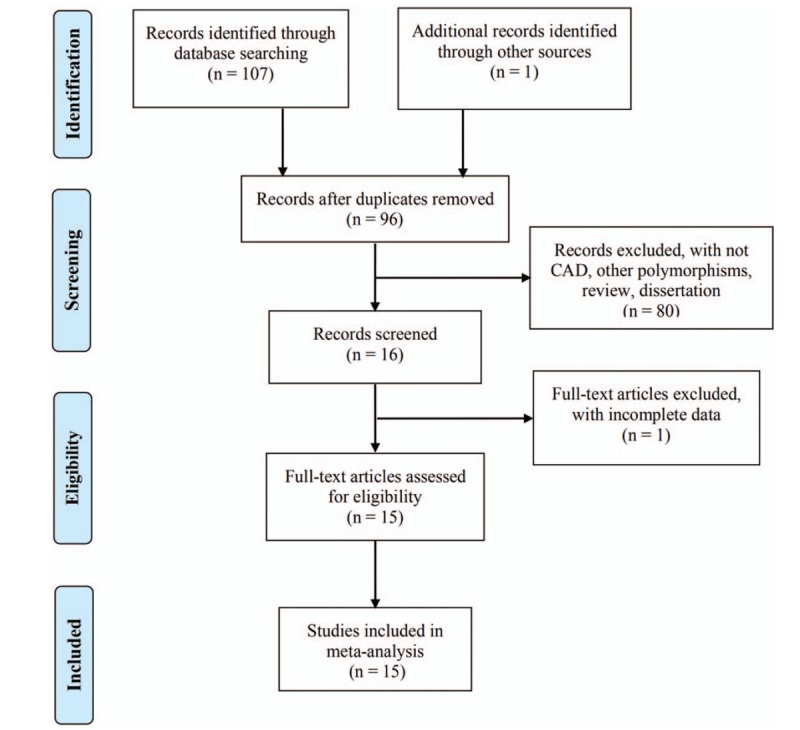
Flow diagram for selection of the included studies.

A total of 107 relevant records were identified through database searching, and 1 was identified manually. The detailed screening process is shown in Figure 1. First, 12 duplicates were removed. Then, 80 records were excluded due to the studied phenotype or genetic locus other than CAD or *ADRB3* Trp64Arg polymorphism, and because of publication types of review, and dissertation, etc. Next, 1 full-text article was found with incomplete genotypic frequency data, and thus removed. Finally, the remaining 15 studies^[7,9,19–31]^ were subjected to quality assessment with NOS criteria, and all were deemed eligible for the following meta-analysis. For the detailed evaluations, see Table 1. The characteristics of the eligible studies are listed in Table 2. All the 15 eligible studies were not significantly deviating from HWE proportions (*P* > .01).

**Table 1:**
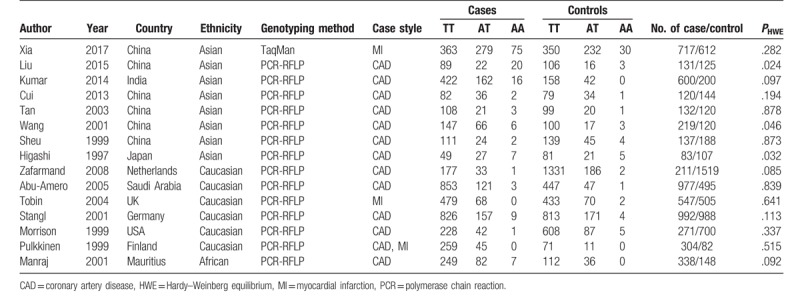
Characteristics of the eligible studies included in the meta-analysis.

**Table 2:**
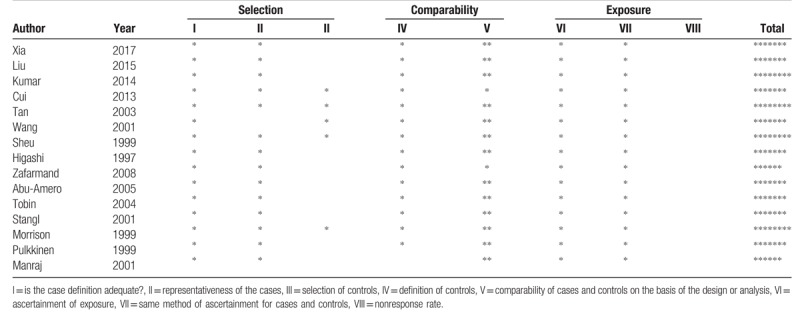
Quality assessment scheme for the eligible studies (Newcastle-Ottawa Scale).

### 3.2 Assessment of the overall risk (across race risk) of *ADRB3* Trp64Arg polymorphism on CAD

A significant association between *ADRB3* Trp64Arg polymorphism and CAD was found in the whole population under allelic, homozygous, heterozygote, dominant, and recessive genetic models (allelic: OR = 1.29, 95%CI 1.09–1.51, *P* = .003; homozygous: OR = 2.43, 95%CI 1.77–3.33, *P* < .001; heterozygous: OR = 1.18, 95%CI 1.02–1.37, *P* = .029; dominant: OR = 1.26, 95%CI 1.07–1.48, *P* = .007; recessive: OR = 2.28, 95%CI 1.67–3.12, *P* < .001) (Table 3).

**Table 3:**
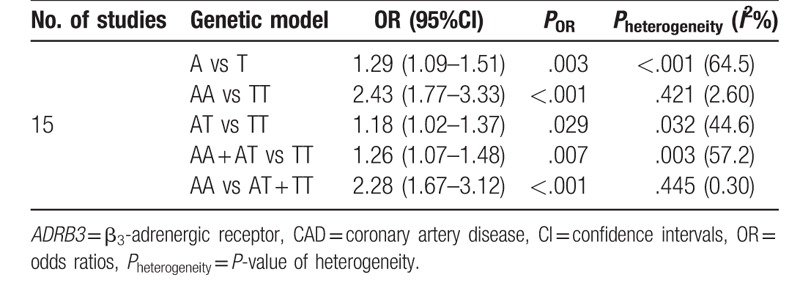
The results for meta-analysis of association between *ADRB3* Trp64Arg polymorphism and CAD risk using the pooled samples without ethnic distinction.

Nevertheless, significant heterogeneity was detected under allelic, heterozygous, and dominant genetic models (allelic: *P*_heterogeneity_ < .001, *I*^2^ = 64.5%; heterozygous: *P*_heterogeneity_ = .032, *I*^2^ = 44.6%; dominant: *P*_heterogeneity_ = .003, *I*^2^ = 57.2%). Careful scrutiny of the forest plots under these genetic models (data not shown) revealed that Asians were largely in opposite direction to Caucasians, judged by the line for the average risk of this polymorphism, thus demonstrating the high level of heterogeneity between the 2 ethnic populations. The only 1 African study showed that the risk of this polymorphism on CAD (OR = 1.20, 95%CI 0.79–1.80) was largely in between Caucasians and Asians, close to the average line.

### 3.3 Assessment of the race-specific risk *ADRB3* Trp64Arg polymorphism by stratified analysis

**Figure 2:**
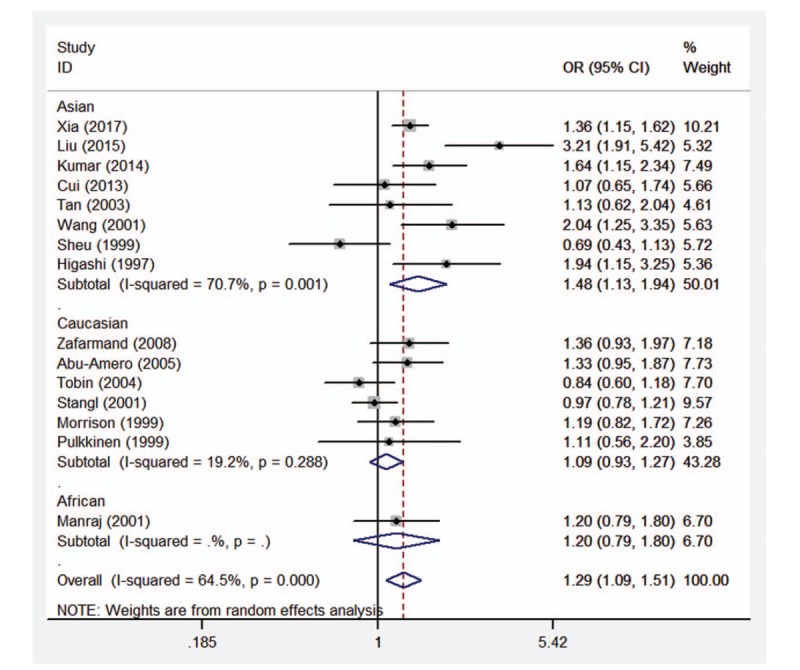
Forest plot of the association between CAD and *ADRB3* Trp64Arg polymorphism under the allelic genetic model (A vs T). *ADRB3* = β_3_-adrenergic receptor, CAD = coronary artery disease.

**Figure 3:**
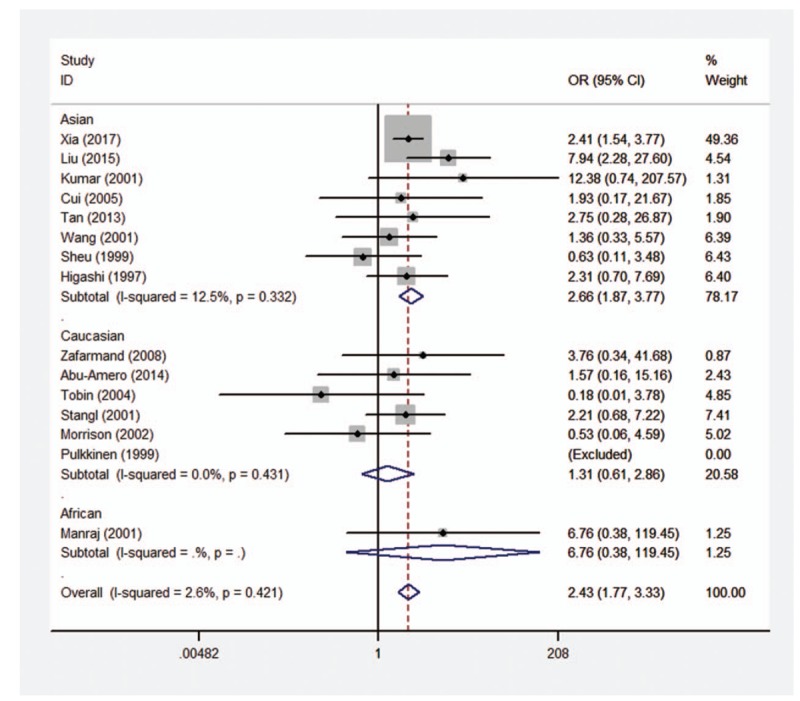
Forest plot of the association between CAD and *ADRB3* Trp64Arg polymorphism under the hemozygous genetic model (AA vs TT). *ADRB3* = β_3_-adrenergic receptor, CAD = coronary artery disease.

**Figure 4:**
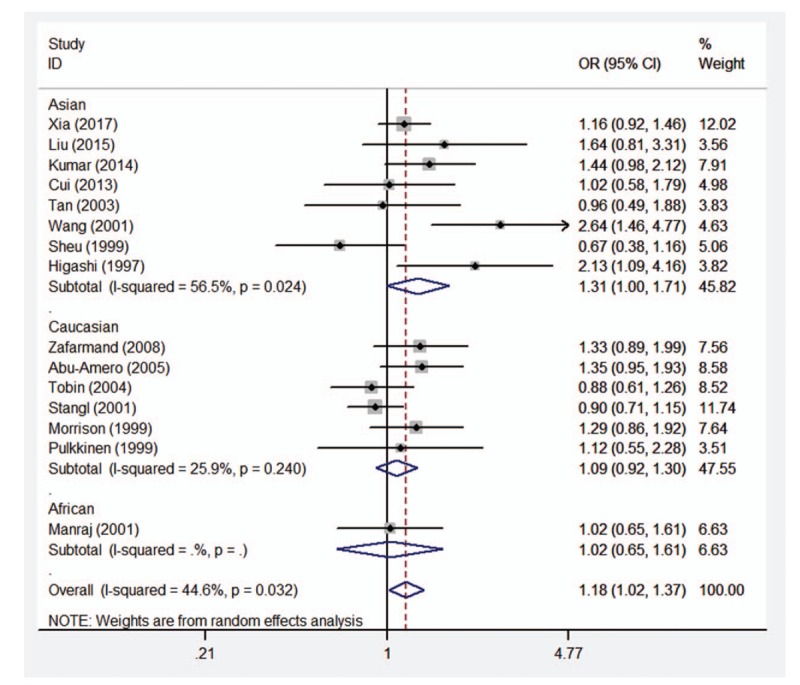
Forest plot of the association between CAD and *ADRB3* Trp64Arg polymorphism under the heterozygous genetic model (AT vs TT). *ADRB3* = β_3_-adrenergic receptor, CAD = coronary artery disease.

**Figure 5:**
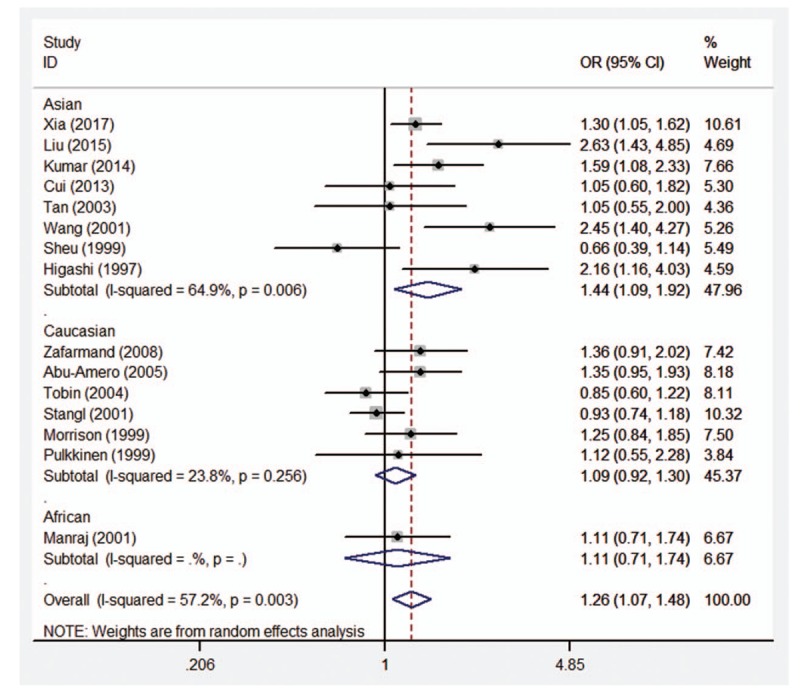
Forest plot of the association between CAD and *ADRB3* Trp64Arg polymorphism under the dominant genetic model (AA + AT vs TT). *ADRB3* = β_3_-adrenergic receptor, CAD = coronary artery disease.

**Figure 6:**
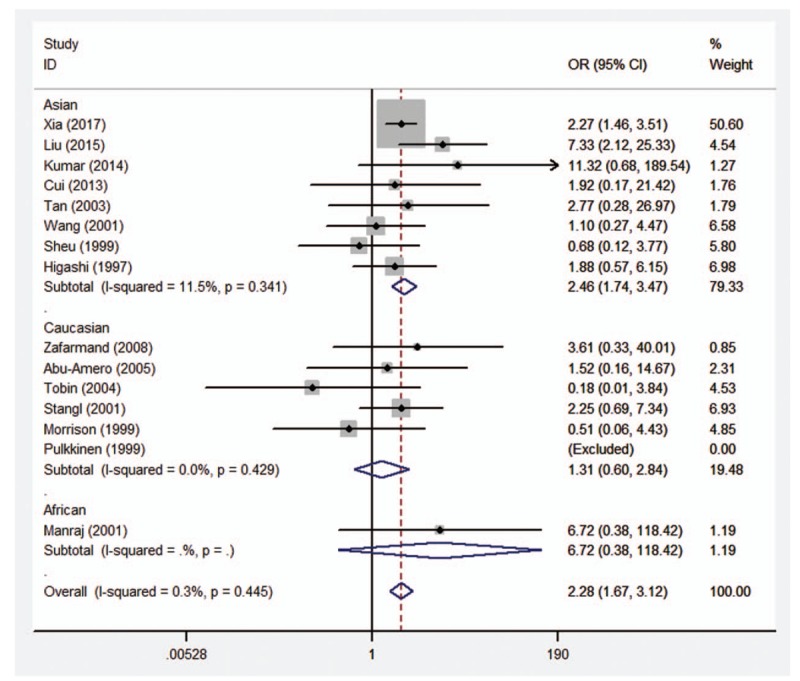
Forest plot of the association between CAD and *ADRB3* Trp64Arg polymorphism under the recessive genetic model (AA vs AT + TT). *ADRB3* = β_3_-adrenergic receptor, CAD = coronary artery disease.

Therefore, the stratified analysis by 3 ethnicities (Caucasians, Asians, and Africans) was performed. The results are shown in Table 4. There were significant association between *ADRB3* Trp64Arg polymorphism and CAD risk in Asian populations under allelic (OR = 1.48, 95%CI 1.13–1.94, *P* = .005), homozygous (OR = 2.66, 95%CI 1.87–3.77, *P* < .001), dominant (OR = 1.44, 95%CI 1.09–1.92, *P* = .011), and recessive (OR = 2.46, 95%CI 1.74–3.47, *P* < .001) genetic models, however no significant association was found in any genetic models in Caucasian population (allelic: OR = 1.09, 95%CI 0.93–1.27, *P* = .290; homozygous: OR = 1.31, 95%CI 0.61–2.86, *P* = .490; heterozygous: OR = 1.09, 95%CI 0.92–1.30, *P* = .333; dominant: OR = 1.09, 95%CI 0.92–1.30, *P* = .308; recessive: OR = 1.31, 95%CI 0.60–2.84, *P* = 2.494) or African population (allelic: OR = 1.20, 95%CI 0.79–1.80, *P* = .394; homozygous: OR = 6.76, 95%CI 0.38–119.45, *P* = .192; heterozygous: OR = 1.02, 95%CI 0.65–1.61, *P* = .916; dominant: OR = 1.11, 95%CI 0.71–1.74, *P* = .641; recessive: OR = 6.72, 95%CI 0.38–118.42, *P* = .193). Forest plots of the association between CAD and *ADRB3* Trp64Arg polymorphism under the 5 genetic models (allelic, homozygous, heterozygous, dominant, and recessive) are shown in Figures 2–6.

**Table 4:**
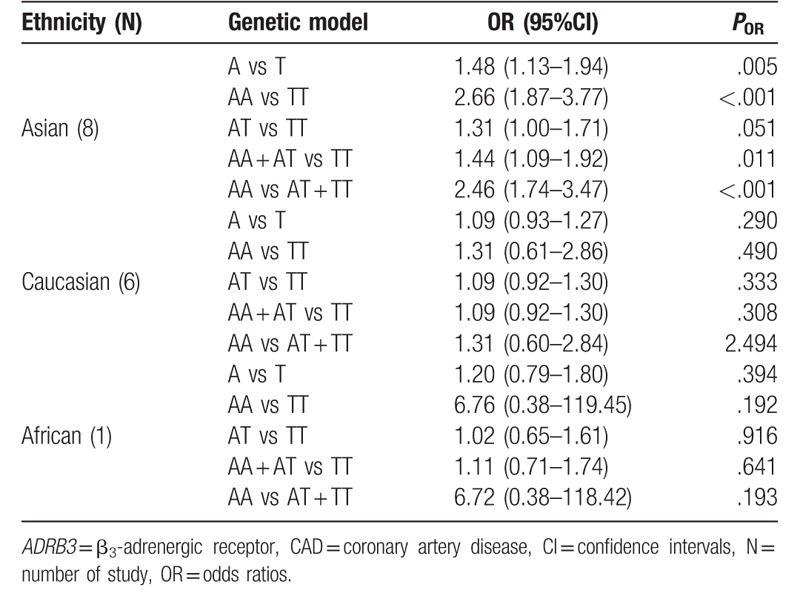
The results for stratified meta-analysis of association between *ADRB3* Trp64Arg polymorphism and CAD risk.

### 3.4 Publication bias

**Figure 7:**
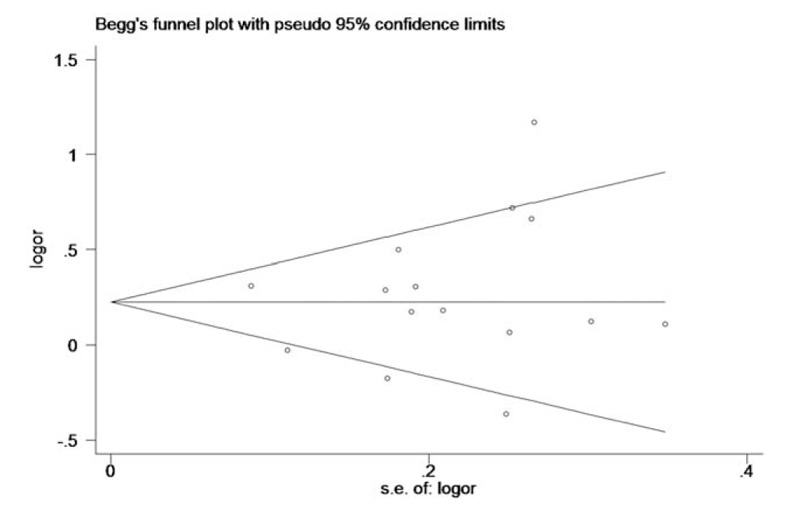
The funnel plot of the association between CAD and *ADRB3* Trp64Arg polymorphism under the allelic genetic model (A vs T). *ADRB3* = β_3_-adrenergic receptor, CAD = coronary artery disease.

Both the Begg's funnel plot and Egger's linear regression test were adopted to evaluate the publication bias of all 15 studies. The funnel plot of the association between *ADRB3* Trp64Arg polymorphism and CAD for A vs T allele is shown in Figure 7. The *P*-value for Begg's test of *ADRB3* Trp64Arg polymorphism A vs T allele was .381, and the *P*-value for Egger's linear regression test was .165. The shape of the funnel plot was symmetrical at large, and there was no statistical significance based on the *P*-values of Begg's test and Egger's linear regression test. The results under other 4 models were very similar (hence data not shown), suggesting no evidence of publication bias among the included studies.

### 3.5 Sensitivity analysis

**Figure 8:**
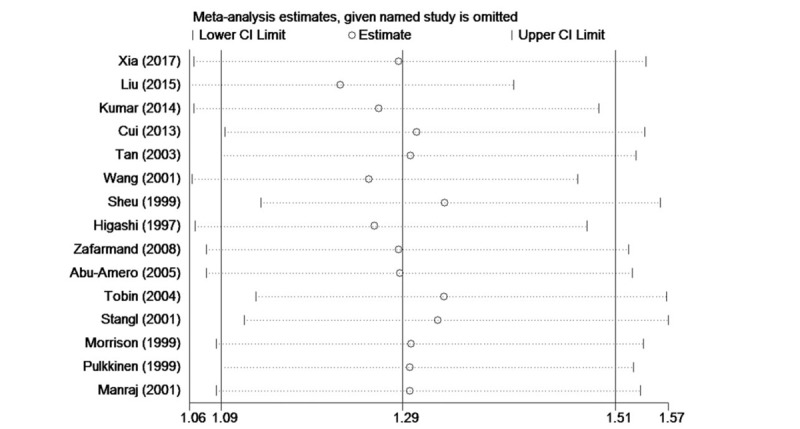
The sensitivity analysis plot of the association between CAD with *ADRB3* Trp64Arg polymorphism under the allelic genetic model (A vs T). *ADRB3* = β_3_-adrenergic receptor, CAD = coronary artery disease.

Finally, sensitivity analysis was performed to assess the influence of individual studies. A leave-one-out procedure (i.e., exclude 1 study each time) was adopted to evaluate the contribution of a single study to the pooled OR value. This sensitivity analysis revealed that none of the studies influenced the pooled OR to any great extent. The leave-one-out OR estimates ranged from 1.25 (1.06–1.47) to 1.33 (1.13–1.56) for A vs T allele (Fig. 8), genotypic OR estimates varied similarly (data not shown). Further the sensitivity analysis by ethnicity gave similar results (data not shown). In short, these sensitivity analyses suggest that the estimates of allelic and genotypic risks obtained in this study were both stable and reliable.

## 4 Discussion

CAD is a complex illness that affects human health and quality of life worldwide. The proportion of deaths from CAD in Asia, particularly China, has also gradually increased.^[32]^ Numerous genetic studies suggested that a large number of genes and their complicated interplays contributed to the development of CAD.^[33–35]^ However, due to various limitations (e.g., presence of genetic heterogeneity, inadequate sample size) inherent in a population based study, single individual study unlikely provides an accurate and robust estimate of genetic risk on CAD. For example, though many studies on the relationship between *ADRB3* gene polymorphism Trp64Arg and CAD have been performed, no conclusion has been reached so far.

Human *ADRB3* gene is located in 8p11.1-p12 and has a full length of 3672 bp, containing 2 exons and 1 intron, encoding 408 amino acids. Previous studies demonstrated that *ADRB3*, beta-3 adrenergic receptor, participated in lipolysis and thermogenesis in adipose tissue. Trp64Arg polymorphism is the only functional variant of *ADRB3* protein. The *ADRB3* Trp64Arg locus acts as a “thrifty” gene and has already been reported associating with obesity,^[36]^ hypertension,^[37]^ diabetes mellitus,^[8]^ insulin resistance,^[38]^ and glycolipid metabolism.^[39]^ Logically, *ADRB3* Trp64Arg locus may play an important role in the pathogenesis of CAD.

Prior to the present study, only 1 meta-analysis of the association between *ADRB3* Trp64Arg locus and CAD had been performed by Zafarmand et al^[9]^ in 2008. Their analysis included 10 studies, and did not find significant association for *ADRB3* Trp64Arg polymorphism and CAD. In the current meta-analysis, more extensive and more recent literature of both English and Chinese had been sought for, and 15 studies were finally included, with 8 Asian studies, 6 Caucasian studies, and 1 African study. In addition to the pooled analysis for assessing the overall risk of *ADRB3* Trp64Arg polymorphism on CAD, a stratified meta-analysis by ethnicity aiming to evaluate the race-specific effect of this locus was conducted. Compared to Zafarmand et al's study, our analyses were more informative and more comprehensive, and therefore our results were more objective and more convincing. Following these meta-analyses, we further evaluated the publication bias of the included studies. Both the roughly symmetrical shape of the funnel plots and the *P*-value of Begg's and Egger's test suggested that there was no evidence of publication bias. Moreover sensitivity analysis also confirmed the stability of our results. Overall, this data analysis revealed a marked difference in genetic diversity among different ethnicity groups. We viewed that the race-specific effect of *ADRB3* Trp64Arg locus on CAD in Asians, but not in Caucasians was well supported, which did not agree very well with a previous meta-analysis that reported no significant association of this locus with CAD in the general population (i.e., without ethnic distinctions). Possible explanations accounting for this inconsistency may be due to genetic heterogeneities across different ethnicities or sampling variance.

In short, our study had several merits. First, we significantly extended sample size, especially including more Asian studies published in Chinese, which rendered our findings more stable and more detailed. Second, we adopted the very strict inclusion and exclusion criteria. All the 15 studies were subjected to the NOS assessment, and had achieved high quality scores of 5 stars or more. Third, in order to fully assess the relationship between this polymorphic locus and CAD, we performed a comprehensive and stratified meta-analysis by ethnicity, under the 5 conventional genetic models, which demonstrated that this locus was of race-specific effect, and the most likely inheritance mode was recessive.

We recognized several potential limitations of this study. First, the searched literature was limited to Chinese and English, which might miss some important studies published in other languages. Second, Asian studies were not further divided by either ethnicity or geographic areas, which may also have slight different genetic diversity. Finally, due to the lack of adequate information regarding the participants’ demographic and epidemiological factors (including age, sex, body mass index, and environmental exposure, etc), we did not perform any stratified analysis regarding these potentially confounding factors, which might bias our statistical estimates.

In conclusion, our results suggested that *ADRB3* Trp64Arg polymorphism confers a race-specific effect to CAD. Stratified analysis by ethnicity revealed that *ADRB3* Trp64Arg polymorphism was significantly associated with CAD in Asians, but not in Caucasians.

## Author contributions

**Conceptualization:** Yingjian Chen, Yuanjun Liao.

**Data curation:** Yingjian Chen, Yuanjun Liao.

**Formal analysis:** Yingjian Chen, Yuanjun Liao, Shengnan Sun, Fan Lin, Rang Li Shujin Lan.

**Resources:** Yuanjun Liao, Xiaolei Zhao, Jiheng Qin, Shaoqi Rao.

**Software:** Yingjian Chen, Yuanjun Liao.

**Writing – original draft:** Yingjian Chen, Yuanjun Liao, Shaoqi Rao.

**Writing – review & editing:** Yingjian Chen, Yuanjun Liao, Shaoqi Rao, Xiaolei Zhao, Jiheng Qin.

Shaoqi Rao: 0000-0001-7809-3700.
